# On the Viability of Video Imaging in Leak Rate Quantification: A Theoretical Error Analysis

**DOI:** 10.3390/s21175683

**Published:** 2021-08-24

**Authors:** Amir Montazeri, Xiaochi Zhou, John D. Albertson

**Affiliations:** 1 Sibley School of Mechanical and Aerospace Engineering, Cornell University, Ithaca, NY 14853, USA; am2774@cornell.edu; 2 School of Civil and Environmental Engineering, Cornell University, Ithaca, NY 14853, USA; xiaochi.zhou@arb.ca.gov

**Keywords:** gas imaging, error analysis, source rate quantification, natural gas, methane emission, remote measurements

## Abstract

Optical gas imaging through multispectral cameras is a promising technique for mitigation of methane emissions through localization and quantification of emissions sources. While more advanced cameras developed in recent years have led to lower uncertainties in measuring gas concentrations, a systematic analysis of the uncertainties associated with leak rate estimation have been overlooked. We present a systematic categorization of the involved uncertainties with a focus on a theoretical analysis of projection uncertainties that are inherent to this technique. The projection uncertainties are then quantified using Large Eddy Simulation experiments of a point source release into the atmosphere. Our results show that while projection uncertainties are typically about 5% of the emission rate, low acquisition times and observation of the gas plume at small distances from the emission source (<10 m) can amount to errors of about 20%. Further, we found that acquisition times on the order of tens of seconds are sufficient to significantly reduce (>50%) the projection uncertainties. These findings suggest robust procedures on how to reduce projection uncertainties, however, a balance between other sources of uncertainty due to operational conditions and the employed instrumentation are required to outline more practical guidelines.

## 1. Introduction

With technological advances in extraction techniques [1], the production of Natural Gas (NG) in the United States underwent a steady increase in the 2010s and reached a new record high in 2019 [2]. Operational and accidental emissions at NG production, processing and transmission facilities release methane, the major component of NG, into the atmosphere, posing risks associated with climate change and health and safety [3]. Therefore, mitigation of emissions has become a top priority in the United States, highlighted by the introduction of periodic leak detection and repair (LDAR) surveys for methane by the U.S. Environmental Protection Agency (EPA) in the 2016 updates to the New Source Performance Standards [4]. In practice, LDAR programs following U.S. EPA’s Method 21 or using optical gas imaging (OGI) are effective for component-level leak detection, however, they are labor and resource intensive, which prevents frequent survey and prompt mitigation efforts. While the focus of LDAR programs has been on leak detection and localization, quantification of emission sources can lead to more effective emission mitigation by prioritizing repair for larger leaks. Furthermore, quantification of small and medium-sized emission sources adds depth to our understanding of emission profiles associated with the NG supply chain that can lead to further mitigation efforts.

In recent years, new measurement systems and technologies have been developed to quantify emissions of methane from equipment and operations with reduced cost and/or improved spatial coverage. Examples include portable methane analyzers [5], open-path laser spectrometers [6], remote sensing of methane from aircraft and satellite [7,8,9], ground-based mobile sensing [10,11], and airborne mobile sensing approaches using unmanned aerial vehicles (UAVs) [12,13,14,15]. Use of portable methane analyzers is an accurate method for quantifying known emission sources, but localization efforts are often labor intensive with the requirement of investigating entire facilities at a slow pace. Open-path laser spectrometers are used to quantify emissions from facilities, but require equipment for wind measurement in addition to knowledge of location of emitting components that necessitates accompanied use of detection methods such as OGI. Satellite and airborne methods allow coverage of large areas and detection of relatively large emission sources (e.g., detection limit for the Airborne Visible/Infrared Imaging Spectrometer—Next Generation is 240 kg CH_4_/day) [9]. However, such high detection limits prevent identification of low- and moderate-emission sources under typical meteorological conditions. Ground-based mobile sensing approaches are useful in quantifying emissions from facilities without offering solutions for component leak localization [16,17]. While the use of UAV systems has been promising for leak detection, localization and quantification, large uncertainties arise from the reliance on lightweight sensors and simplistic dispersion models [12,14]. It is worth noting that many non-invasive approaches including transient test-based techniques (TTBTs) have shown success in leak detection and quantification in water systems [18,19,20]. Future studies that employ these methods in natural gas systems could shed a light on their applicability in estimating methane emissions.

One newly developed technique that allows for quantification and localization is ground-based remote sensing via gas imaging cameras [21,22,23]. Gas imaging includes capturing video images of methane plumes in the environment to quantify emission rates. Briefly, this technique involves the comparison of the at-sensor radiant energy in the IR part of the electromagnetic spectrum in the presence and absence of the gas plume [23,24]. This difference in radiance is then related to the depth integrated concentration of the gas (also known as the concentration-path length) in ppm×m through the use of Beer-Lambert law and the temperature contrast between the gas and the background scene. This technique shows promise as it offers high spatiotemporal resolution in mapping gas concentrations as well as possibility of automation and continuous monitoring of sites. In addition, ancillary equipment for wind measurement is not required, since with high frequency imaging (e.g., >1 Hz) gas velocities inside the plume can be approximated by tracking plume features in consecutive images using velocimetry algorithms such as minimum quadratic differences [25], cross-correlation between consecutive images [26], and block-matching [21]. With the measured methane concentration and the estimated flow velocity, the emission rates can be computed based on the principle of mass balance. However, a systematic analysis focusing on the uncertainty of such estimates has been lacking in the literature.

The effectiveness of gas imaging techniques in quantifying unknown leak rates is tied to the level of uncertainty in leak quantification. Furthermore, lower uncertainties in leak rate quantification lead to lower false alarm rates and promote effective mitigation of emissions and reduced costs [27]. An in-depth understanding of the sources of uncertainty is essential for increasing the accuracy and precision of leak quantification. To this end, we divide the uncertainties into the following categories: (1) instrumentation, (2) operational and (3) two-dimensional projection uncertainties. Lower uncertainties can generally be achieved through technological advances in equipment and instrumentation. For example, increased spatial and temporal resolution of imaging cameras translate into lower uncertainty in concentration measurement and velocity estimation which in turn leads to lower total uncertainty in leak quantification [27]. Instrumentation uncertainties are usually reported by manufacturers and have been previously studied in detail [21,22,26,28]. Meanwhile, the transformation of concentrations and velocities into emission rates is also an uncertain process. The uncertainties of this transformation process can be due to operating conditions (operational uncertainty) or arise from approximating the three-dimensional (3D) methane plume, using a two-dimensional (2D) view as seen by the camera (projection uncertainties). Operating conditions such as distance between camera and leak, background temperature, changes in wind speed and direction can affect uncertainty levels by affecting detection capabilities of the cameras (e.g., the minimum leak size that can be detected) and have been previously investigated for leak detection through simulations and experiments [29,30]. On the other hand, projection uncertainties have been largely ignored in the literature. It is worth noting that while operating conditions and instrumentation uncertainties can affect the magnitude of projection uncertainties, projection uncertainties are ubiquitous irrespective of other uncertainties. In other words, even with perfect equipment and algorithms accurately measuring concentrations and velocities, projection uncertainties will still be present.

In this paper, we formulate the projection uncertainties related to the transformation of gas imaging measurements into emission rates through a rigorous theoretical analysis that couples mass balance and spatial Reynolds decomposition. Our analysis is first carried out through comparison of a 3D instantaneous plume transport model and its 2D projection which models an emission scene as observed through the lens of a camera. Our analysis divides the projection uncertainties into two distinct uncertainty expressions. These two expressions are then quantified and compared against each other using Large eddy simulations (LES) of a point source plume dispersion under neutral atmospheric conditions. Furthermore, the effects of acquisition time and downwind distance from leak on projection uncertainties are quantified. Finally, we discuss the implications of these results on the viability of gas imaging techniques on leak rate quantification.

It is worth noting that this article focuses on fugitive emissions of methane due to the recent incentives for accurate quantification, namely the DOE ARPA-E’s Methane Observation Networks with Innovative Technology to Obtain Reduction (MONITOR) program [31], however, our theoretical analysis stands true for video observations of any release of a conserved scalar into the environment.

## 2. Theory

To evaluate the projection uncertainties in leak rate quantification through gas imaging, we need to derive expressions for the error terms involved. To this end, in Section 2.1 we describe an instantaneous view of plume transport and use it to formulate an exact solution to a point source leak rate problem. This formulation has been previously used to characterize point sources through mobile sensor data with details available in [32]. In Section 2.2, we introduce a 2D projection of the transport formulation to model images captured in gas imaging experiments. We use these 2D projections to formulate an approximate solution to the leak rate problem. The difference between the exact and approximate solutions explicitly describes the projection uncertainties present in leak rate quantification as shown in Section 2.3.

### 2.1. Instantaneous 3D View of Plume Transport

**Figure 1:**
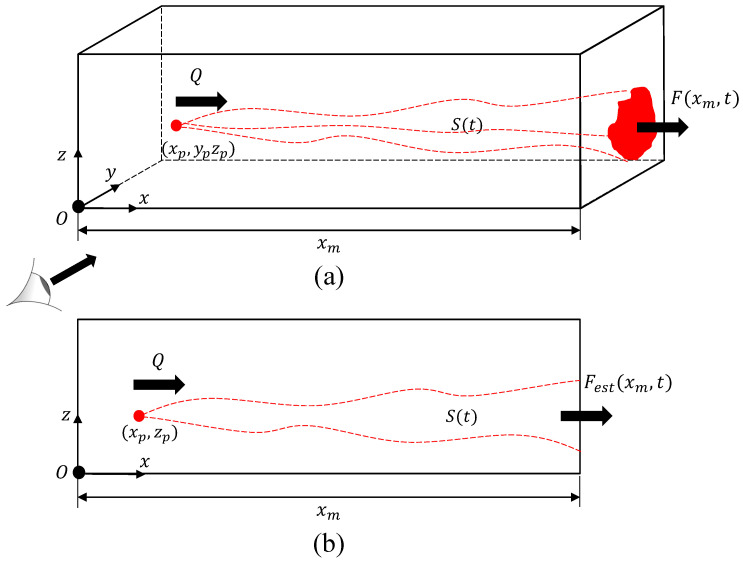
A control volume containing a continuous source with a mass flow rate of Q located at $\left(x_{p},y_{p},z_{p}\right)$ in (**a**) a three-dimensional view, with a cross-plane view of the plume mass flow rate, $F(x_{m},t)$ at downwind distance, $x_{m}$ and (**b**) a two-dimensional snapshot modeling an image obtained via gas imaging leading to an estimate of the mass flow rate, $F_{est}\left(x_{m},t\right)$ at downwind distance, $x_{m}$.

We consider the release of a gas (e.g., methane) from a point source into the environment. The release is happening in the surface-layer of the atmospheric boundary layer (ABL) where the assumptions of stationary and horizontally homogeneous turbulence hold [33]. Without loss of generality, we assume that the x-axis of the coordinate system is directed along the mean wind, and we refer to the *y* and *z* directions as “depth” and “height” directions, respectively. This setup is shown in Figure 1a, with the point source located at $\left(x_{p},y_{p},z_{p}\right)$. In this setup, u,v and *w* are defined as velocity components in x,y and *z* directions, respectively. A control volume is defined starting from the origin *O* and extending in the three principal directions up to downwind sampling positions $x_{m}$, from $y_{min}$ to $y_{max}$ in depth, and $z_{min}$ to $z_{max}$ vertically. The control volume contains the source and is defined such that the plume generated from the point source only exits the face on the y−z plane at $x=x_{m}$. Conservation of mass states that the source rate (mass per time), *Q* can be expressed as

(1) $$Q=F\left(x_{m},t\right)+\frac{dS(t)}{dt},$$

where S(t) is total mass of the emitted gas in the control volume, *t* is time, and $F(x_{m},t)$ is the mass flow rate out of the control volume.

The total mass of the emitted gas in the control volume at any time, *t*, is calculated by integrating the above-ambient concentration over the full control volume as follows

(2) $$S\left(t\right)=\int_{0}^{x_{m}}\int_{y_{min}}^{y_{max}}\int_{z_{min}}^{z_{max}}c\left(x,y,z,t\right)dzdydx,$$

where *c* is the above-ambient gas concentration. In the ABL, the flow is highly turbulent so that molecular diffusion can be ignored relative to turbulent transport [34]. Therefore, the mass flow rate exiting the downwind face of the control volume is related to the gas concentration and velocity as

(3) $$F\left(x_{m},t\right)=\int_{L_{z}}\int_{L_{y}}c\left(x_{m},y,z,t\right)u\left(x_{m},y,z,t\right)dydz,$$

where $L_{y}$ and $L_{z}$ are the plume depth and height, respectively.

Equations (1)–(3) allow for quantification of the source rate with knowledge of the gas concentration and plume velocity as functions of space and time. These expressions are considered the benchmark to which we will compare other formulations to evaluate their intrinsic uncertainties. Note that the precise local gas concentrations and velocities are not readily available through gas imaging techniques; therefore, we simulate the image sampling process in a 2D model framework so as to quantify the truncation errors introduced by the measurements and analytics.

### 2.2. 2D Modeling of Instantaneous Plume Transport

In order to model the images of plume transport, the control volume introduced above is projected such that the y-axis is collapsed and only the *x* and *z* principal directions are resolved (Figure 1b). In this scenario, it is not possible for a camera to obtain the velocity and concentration variations with depth (i.e., variations in y-direction). Instead, a depth-integrated concentration profile is observed [22,23], which is denoted by $c^{y}$ and defined as

(4) $$c^{y}\left(x,z,t\right)=\int_{L_{y}}c\left(x,y,z,t\right)dy.$$

Equation (4) can be utilized to show that in the 2D model the total mass of the emitted gas within the control volume S(t) can be evaluated exactly.

To calculate the mass flow out of the control volume, the velocities inside the plume are also required. In practice, these velocities are measured by employing optical velocimetry algorithms [21,25,26] that track the depth-integrated concentration profiles over consecutive images. The mass flow out of the control volume can be estimated using these *inferred velocity profiles* from plume tracking, labeled $u_{i}\left(x_{m},z,t\right)$, and the depth integrated concentration profiles as follows

(5) $$F_{est}\left(x_{m},t\right)=\int_{L_{z}}c^{y}\left(x_{m},z,t\right)u_{i}\left(x_{m},z,t\right)dz,$$

where $F_{est}$ is the estimated outward mass flow. A detailed discussion on the possibilities for the inferred velocity is presented in Section 4.

### 2.3. Projection Uncertainty Formulation

The projection uncertainty associated with the 2D projection of the plume can be formulated by comparing the leak rate quantification procedures of Section 2.1 and Section 2.2. We continue the analysis under the assumption that instrumentation uncertainties are negligible, meaning that the depth-integrated concentrations measured through gas imaging are without significant error. With this assumption, S(t) can be calculated exactly through the 2D measurement inference algorithm. Therefore, the projection uncertainties in quantifying the leak rate are solely dependent on the difference between *F* and $F_{est}$.

The relationship between *F* and $F_{est}$ can be written explicitly by applying Reynolds decomposition to the dependent variables (*u* and *c*) and decomposing them into a spatial mean and a fluctuating part, e.g., for the velocity, $u\left(x,y,z,t\right)=\bar{u}\left(x,z,t\right)+u^{\prime }\left(x,y,z,t\right)$. Here, $\bar{u}$ denotes the depth-averaged velocity measured across the depth of the plume, and $u^{\prime }$ is the corresponding fluctuating velocity. This decomposition directly leads to the following results

(6) $$\begin{array}{cc} \int_{L_{y}}u^{\prime }\left(x,y,z,t\right)dy & =0, \end{array}$$

(7) $$\begin{array}{cc} \int_{L_{y}}c^{\prime }\left(x,y,z,t\right)dy & =0, \end{array}$$

(8) $$\begin{array}{cc} \int_{L_{y}}\;\bar{c}\left(x,\;z,\;t\right)\;dy & =c^{y}\left(x,z,t\right). \end{array}$$

For simplicity of notation, x,y,z,t will be dropped for the remainder of this section.

By utilizing Reynolds decomposition, a relationship between *F* and $F_{est}$ can be rigorously derived. First, we apply the decomposition to *u* and *c* to rewrite *F* as follows

(9) $$\begin{array}{cc} F(x_{m},t) & =\int_{L_{z}}\int_{L_{y}}cudydz=\int_{L_{z}}\int_{L_{y}}\left(\bar{c}+c^{\prime }\right)\left(\bar{u}+u^{\prime }\right)dydz\\ \; & =\int_{L_{z}}\int_{L_{y}}\left(\bar{c}.\bar{u}+\bar{c}u^{\prime }+\bar{u}c^{\prime }+c^{\prime }u^{\prime }\right)dydz\\ \; & =\int_{L_{z}}\bar{u}c^{y}dz+\int_{L_{z}}\int_{L_{y}}c^{\prime }u^{\prime }dydz \end{array}$$

where Equations (6)–(8) are used to simplify terms along the way. Subtracting Equation (5) from Equation (9) yields the difference between *F* and $F_{est}$

(10) $$F\left(x_{m},t\right)-F_{est}\left(x_{m},t\right)=\int_{L_{z}}\left(\bar{u}-u_{i}\right)c^{y}dz+\int_{L_{z}}\int_{L_{y}}c^{\prime }u^{\prime }dydz.$$

With the assumption that instrumentation uncertainties are negligible, the right hand side of Equation (10) describes the projection uncertainties present in leak rate quantification via gas imaging, since they are caused by using a 2D projection of the plume to approximate the mass flow rate. We note that although such an assumption is not valid in practice, it allows us to isolate and estimate the projection uncertainties. In practical applications, the projection uncertainties should be added to other uncertainty estimates for a better quantification of the total uncertainties. The first integral in Equation (10) scales with the difference between the true depth-averaged velocity and the inferred velocity estimate from the 2D image analysis. Therefore, prediction of the scale of this velocity difference under typical application conditions indicates the importance of the first term. The second integral describes the covariance of velocity and concentration fluctuations. Hereafter, we will refer to the first integral as the “mean velocity error term” and the second integral will be referred to as the “covariance error term”. To quantify the significance of each error term compared to the leak rate, we define the “normalized covariance error” ($\Phi_{c}$) and the “normalized mean velocity error” ($\Phi_{u}$) as follows

(11) $$\begin{array}{cc} \Phi_{c}\left(x_{m},t\right) & =\frac{\int_{L_{z}}\int_{L_{y}}c^{\prime }u^{\prime }dydz}{Q}, \end{array}$$

(12) $$\begin{array}{cc} \Phi_{u}\left(x_{m},t\right) & =\frac{\int_{L_{z}}\left(\bar{u}-u_{i}\right)c^{y}dz}{Q}. \end{array}$$

Before exploring the scale of the error terms by analyzing a dataset acquired through Large Eddy Simulations (LES), we will discuss a number of considerations related to the inferred velocity.

### 2.4. Inferred Velocity Considerations

The mean velocity error term is a function of the inferred velocity, ($u_{i}$), which is dependant on the operational conditions and the velocimetry technique used to infer the velocity from the gas imaging. There are numerous possibilities for the inferred velocities, among which we analyse three possible cases:

#### Case 1: Concentration weighted average velocity (ideal case)

In this scenario, the inferred velocity is computed as a concentration weighted average velocity (also referred to as plume-weighted advection velocity) [32]:

(13) $$u_{i}^{\left(1\right)}\left(x_{m},z,t\right)\equiv \frac{\int_{L_{y}}ucdy}{\int_{L_{y}}cdy},$$

where the superscript is used to show that these expressions are only valid for the corresponding case of the discussion regarding the mean velocity error term. Equation (13) can then be used to rewrite the mass flow out of the control volume

(14) $$F\left(x_{m},t\right)=\int_{L_{z}}\int_{L_{y}}cudydz=\int_{L_{z}}u_{i}^{\left(1\right)}\int_{L_{y}}cdydz=\int_{L_{z}}u_{i}^{\left(1\right)}c^{y}dz=F_{est}^{\left(1\right)}\left(x_{m},t\right).$$

Therefore, with the concentration weighted average velocity as the inferred velocity, the mean velocity error term cancels out the covariance term in the projection uncertainty calculations, allowing for the mass flow out of the control volume to be computed exactly. In practice, this case in unlikely to be achieved, hence we continue by establishing an upper bound for the normalized mean velocity error.

#### Case 2: Maximum difference velocity (upper bound case)

To compute an upper bound for the normalized mean velocity error, the inferred velocity can be expressed as follows

(15) $$u_{i}^{\left(2\right)}\left(x_{m},z,t\right)\equiv \bar{u}+\max\left\{\left|u-\bar{u}\right|:y\in y_{plume}\right\},$$

where $y_{plume}$ corresponds to the interval of length $L_{y}$ where the plume at $\left(x_{m},z\right)$ is instantaneously located.

#### Case 3: Maximum concentration velocity

A plausible estimation for the inferred velocity is the velocity of a portion of the plume that has the highest concentration. In a gas flow, clumps of higher concentration contribute more to the measured depth-integrated concentrations than other parts of the plume. Therefore, it is expected that velocimetry techniques infer the gas velocity by tracking these highly concentrated clumps [28]. As a result, another possibility for inferred velocity can be defined as follows

(16) $$u_{i}^{\left(3\right)}\left(x_{m},z,t\right)\equiv u\left(x_{m},\underset{y}{arg max}c\left(x_{m},y,z,t\right),z,t\right).$$

## 3. Large Eddy Simulation Data

Large Eddy Simulation is used to create a virtual test site for simulation of the dispersion of a passive scalar (e.g., methane) in the surface layer of the ABL. The LES turbulent modeling is particularly useful for simulating high-Reynolds number flows in the ABL. The LES code used in this study has been utilized and validated in numerous studies [35,36,37,38,39]. In brief, the code numerically solves the resolved Navier-Stokes and mass conservation equations on a Cartesian grid while the unresolved (sub-grid) dynamics are closed in terms of the resolved scales [36].

LES has been previously used as a realistic proxy for the space-time evolution of plumes in turbulent near-neutral environments [39,40,41,42]. Therefore, in this study, methane release from a point source is simulated under near-neutral turbulent conditions in an unobstructed flat homogeneous terrain.

The virtual site was set up with 0.469 m horizontal and 0.188 m vertical grid resolution with a total simulation domain size of 60 m in *x* (along-wind) and *y* (crosswind) directions and 15 m in *z* (vertical) direction (128×128×80 spatial resolution). The virtual site was constructed to resemble the Methane Emissions Technology Evaluation Center (METEC) well pads, a facility funded through the ARPA-E’s MONITOR program and built to provide a location that models natural gas production sites. In the virtual site, the source is located at a height of 2.25 m to match the average height of a typical leak as modeled in the METEC facilities. A 30-min spin-up period found by trial and error was implemented to allow the simulated turbulence to reach a stationary state. In this case, the average and standard deviations of the wind and scalars approach a constant value [39]. For the analysis to follow, 5 y−z intersects are created at non-dimensional downwind distances normalized by the source height $\left(x_{m}/z_{p}\right)$ of approximately 2, 3, 4, 8 and 12, on which the instantaneous velocities and concentrations are sampled (recorded) at a frequency of 1 Hz for the duration of 15 min. A summary of parameters used in the LES is presented in Table 1.

## 4. Results and Discussion

In this section, we utilize the LES dataset to estimate the scale of the covariance and velocity terms as functions of sampling distance to leak and sampling duration.

### 4.1. Covariance Error Term

**Figure 2:**
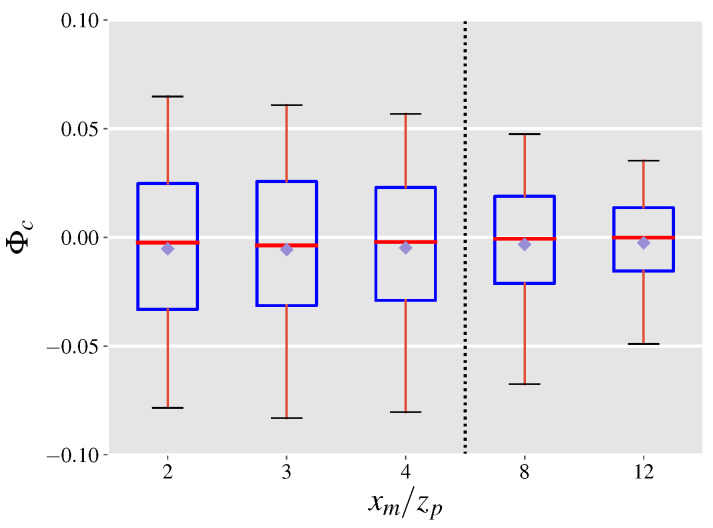
Distributions of the normalized covariance error shown at 5 downwind y−z intersects from the emission source measured for every saved snapshot from the LES. Box and whiskers plots show the median (red), 25th and 75th percentile (blue), the 5th and 95th percentile (black), and the mean (purple diamond) values of each distribution.

To observe the significance of the covariance term the LES dataset is employed. For each snapshot (saved at a frequency of 1 Hz), the normalized covariance error is calculated at the y−z intersects located downwind of the emission source. Box plots of populations of $\Phi_{c}$ calculated at each of the 5 y−z intersects distinguished by their downwind distance from the source, are presented in Figure 2. The figure shows that at all downwind distances from the source the covariance error term is almost always less than 10% of the leak rate. Further, the significance of the covariance term drops as the downwind distance from the source is increased. A possible explanation for this result is through the assumption of local isotropy at the length scale of the plume. One consequence of local isotropy is the vanishing of all correlations between velocity components and scalars [43] leading to small values for $\Phi_{c}$. Moreover, the drop in $\Phi_{c}$ with distance is due to the fact that at larger distances, concentration fluctuations from the mean become smaller as the plume widens, while the velocity fluctuations stay relatively constant. Meanwhile, the smaller than zero median and mean values suggest that the covariance error term is in a direction opposite to the total mass flow. This finding can be understood by noting that a higher velocity compared to the mean can move the plume and lead to lower local concentrations leading to observing opposite signs for $u^{\prime }$ and $c^{\prime }$ on average.

**Figure 3:**
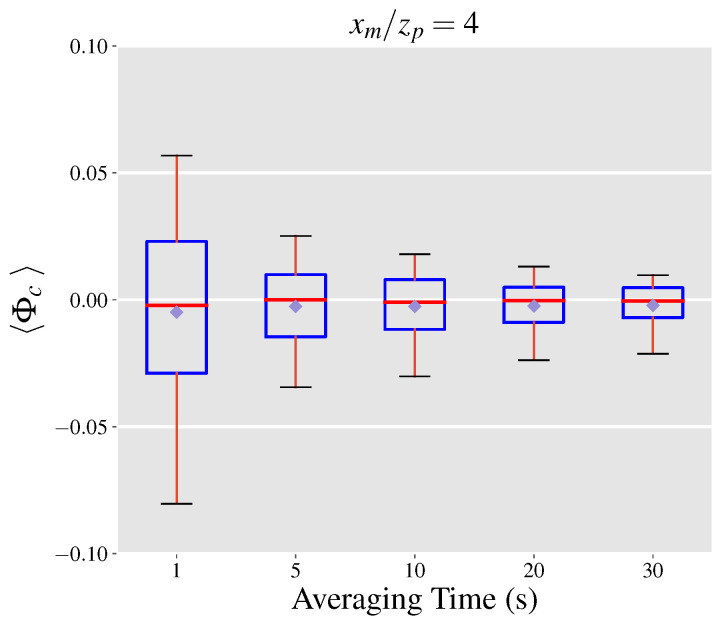
Effect of time-averaging on $\Phi_{c}$ at a normalized downwind distance of $x_{m}/z_{p}=4$. Box and whiskers plots show the median (red), 25th and 75th percentile (blue), the 5th and 95th percentile (black), and the mean (purple diamond) values of each distribution.

The tall whiskers in the box plots in Figure 2 indicate that in a single snapshot, $\Phi_{c}$ can take values in a relatively large interval. Therefore, it is plausible that mass flow rates be computed using multiple snapshots taken over a period of time, highlighting the effect of time-averaging on $\Phi_{c}$. Figure 3 depicts the effect of time averaging on $\Phi_{c}$ for a normalized downwind distance of $x_{m}/z_{p}=4$. In this figure, the y-axis is labeled by $\langle \Phi_{c}\rangle$ to indicate a time-averaged parameter, where the angle brackets denote time-averaging. It can be seen that longer time-averages reduce $\Phi_{c}$ indicating that even short averaging times on the order of tens of seconds can lead to a substantial decrease in the significance of the covariance error term and therefore the projection uncertainty. It is worth noting that a single well-defined wind direction (e.g., <$10^{\circ }$ variation in the mean wind direction) is necessary for accurate velocity and concentration measurements and therefore emission estimates. Moreover, the chance of observing larger variations in wind direction increases as acquisition times are increased. Thus, averaging times longer than 30 s are not shown in Figure 3, even though they lead to further decreases in the normalized covariance error.

### 4.2. Mean Velocity Error Term

**Figure 4:**
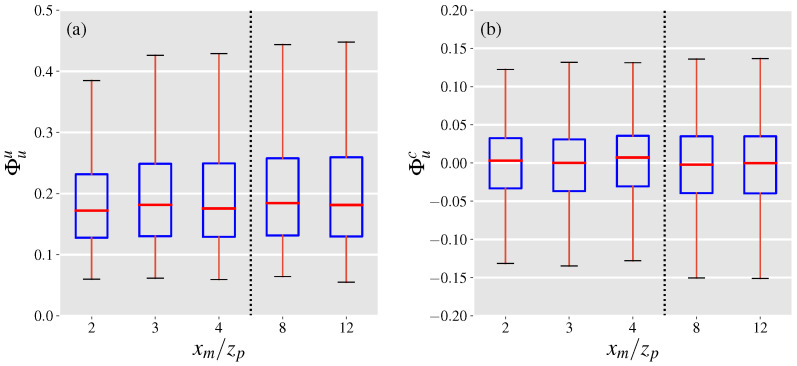
Distributions of (**a**) the upper bound of the normalized mean velocity error and (**b**) the normalized mean velocity error based on using the maximum concentration velocity as the inferred velocity at 5 downwind y−z intersects from the emission source. Box and whiskers plots show the median (red), 25th and 75th percentile (blue), and the 5th and 95th percentile (black) values of each distribution.

The normalized mean velocity error is quantified using the LES data in a similar manner to $\Phi_{c}$. We use $\Phi_{u}^{u}$ to refer to the upper bound of the normalized mean velocity error, which is computed by employing Equation (15) to compute the normalized mean velocity error. Boxplots of populations of $\Phi_{u}^{u}$ at each of the y−z intersects are illustrated in Figure 4a.

**Figure 5:**
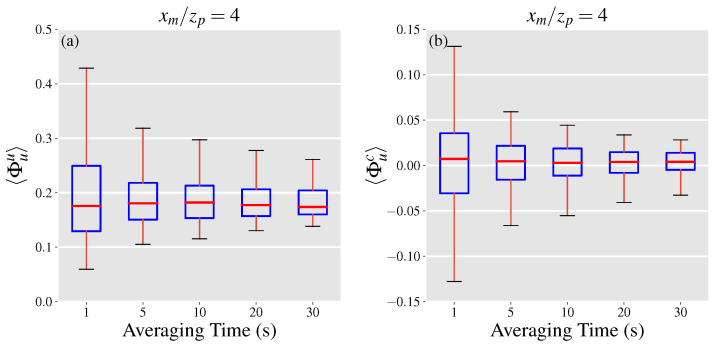
Effect of time-averaging on (**a**) $\Phi_{u}^{u}$ and (**b**) $\Phi_{u}^{c}$ at a normalized downwind distance of $x_{m}/z_{p}=4$. Box and whiskers plots show the median (red), 25th and 75th percentile (blue), and the 5th and 95th percentile (black) values of each distribution.

As the distance from the source is increased, the plume becomes wider, hence we expect the difference term, $\left(\bar{u}-u_{i}^{\left(2\right)}\right)$, to grow. However, as the plume grows wider through diffusion the depth-integrated concentrations at each height are decreased. As a result, Figure 4a shows that at closer distances to the source the rate of increase in $\Phi_{u}^{u}$ is faster compared to larger distances, with $\Phi_{u}^{u}$ almost staying constant between $x_{m}/z_{p}$ of 8 and 12. The effect of time averaging on $\Phi_{u}^{u}$ is presented in Figure 5a highlighting that the range of uncertainties significantly drops as the averaging times are increased in a similar manner to the normalized covariance error. Further, the median value for $\Phi_{u}^{u}$ is under 0.20 which is a promising result for leak quantification using gas imaging based on current standards [31], given that this is the worst case scenario for the inferred velocity.

We use $\Phi_{u}^{c}$ to refer to the normalized mean velocity error computed with $u_{i}^{\left(3\right)}$ as the inferred velocity with boxplots of populations of $\Phi_{u}^{c}$ at 5 y−z intersects illustrated in Figure 4b. In this scenario, the population distribution of $\Phi_{u}^{c}$ becomes wider with increasing distances from the source before reaching a plateau at normalized distances of 8 and 12 in a similar manner to $\Phi_{u}^{u}$. For all considered distances, while the mean velocity error term can reach up to 15% of the leak rate in value, the most likely scale of the error is between ±3 percent. Moreover, the median value of the distributions is within 1% of zero, suggesting that high acquisition times would result in minimal mean velocity errors. Figure 5b shows the effect of averaging time on the population distribution of $\Phi_{u}^{c}$ at $x_{m}/z_{p}=4$, highlighting a significant drop of 50% in the width of the distribution (according to the 5–95 percentiles) when the averaging time is increased to 5 s. This finding alongside the effect of averaging times on the magnitude of the covariance error term, underlines the importance of longer acquisition times in order to reduce the projection uncertainties related to leak quantification. A more formal investigation of the total projection uncertainty is discussed in the next section.

### 4.3. Total Projection Uncertainty

**Figure 6:**
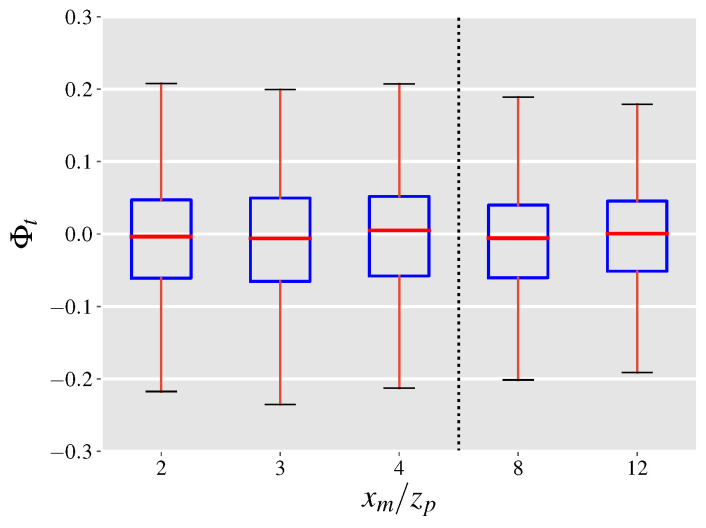
Distributions of the normalized projection uncertainty shown at 5 downwind y−z intersects from the emission source. Box and whiskers plots show the median (red), 25th and 75th percentile (blue), and the 5th and 95th percentile (black) values of each distribution.

**Figure 7:**
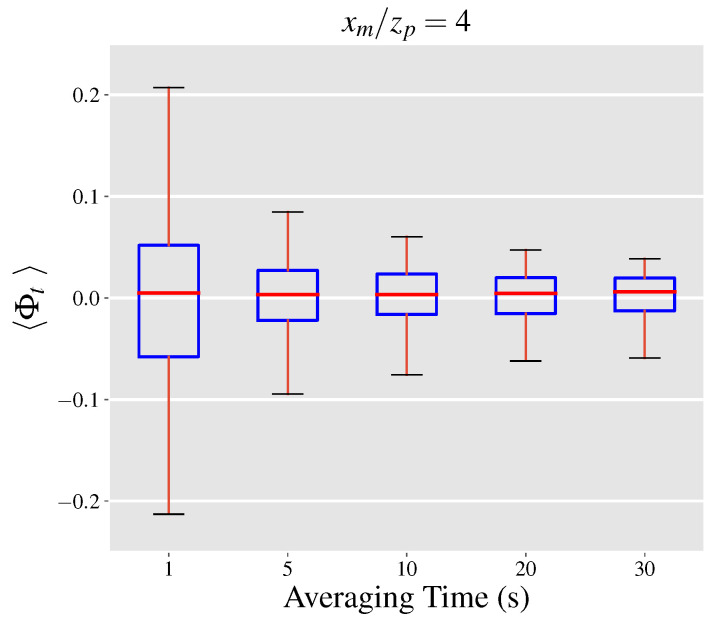
Effect of time-averaging on $\Phi_{t}$ at a normalized downwind distance of $x_{m}/z_{p}=4$. Box and whiskers plots show the median (red), 25th and 75th percentile (blue), and the 5th and 95th percentile (black) values of each distribution.

Here, we define the normalized projection uncertainty as the ratio of the total projection uncertainty to the leak rate. By this definition, the normalized projection uncertainty ratio, denoted by $\Phi_{t}$, can be computed by the addition of the normalized covariance and mean velocity errors. We use the inferred velocity from case (3) above to estimate $\Phi_{t}$, with population boxplots depicted in Figure 6. The results indicate that the projection uncertainty typically lies within 5% of zero and drops as the outgoing surface of the control volume is constructed further away from the point source at $x_{m}/z_{p}$ of 8 and 12. However, the reduction in the uncertainty range does not occur monotonically and the range only slightly varies between normalized distances of 2, 3 and 4. Generally, the covariance error term accounts for at least a quarter of the total projection uncertainty depending on the downwind distance of observations. Increasing the averaging time leads to narrower distributions of $\langle \Phi_{t}\rangle$ as presented in Figure 7 in a similar manner to the normalized covariance and mean velocity errors. A noteworthy observation is the diminishing returns of increasing the averaging time from 20 to 30 s with a relative drop of 17% (absolute drop of 0.02) compared to a relative drop of 54% (absolute drop of 0.22) when the averaging time is increased to from 1 to 5 s.

With the projected uncertainties being smaller at farther distances from the point source, it may seem desirable to observe gas plumes far downstream of the point source for the purpose of leak quantification (especially when long acquisition times are not possible). In practice however, the plume may be difficult to detect and quantify at large distances away from the source due to detection limits of the gas imaging instrument [29]. On the other hand, in addition to higher projection uncertainties, imaging close to the point source can lead to underestimation if the saturation limit of the instrument in observing the depth-integrated concentrations is reached. Therefore, a plausible approach in leak rate estimation would be to employ several control volumes with control surfaces located at varying distances from the point source. The estimated leak rates from the control volumes can be averaged after removing the outliers created due to detection and saturation limits, to compute a final leak rate estimate. Moreover, the acquisition time should be as long as the wind conditions allow, since longer acquisition times are translated into lower projection errors.

## 5. Summary and Conclusions

In this paper, we presented an approach for expressing and quantifying the projection uncertainties in estimating fugitive source emission rates through gas imaging techniques. The projection uncertainties arise from observing the dispersion of a 3D plume in the atmosphere through 2D video images. We developed a theoretical analysis that led to two separate terms associated with the projection uncertainties, the covariance and mean velocity error terms. A simulated dataset generated through Large eddy simulations was used to quantify the significance of each of the projection uncertainty terms under varying imaging constraints of acquisition time, and downwind distance from the leak source.

We found that low acquisition times and instantaneous estimates of the leak rate are prone to high projection uncertainties that can amount up to 20% of the emission rate. However, the typical projection uncertainty is expected to be between ±5 percent highlighting the potential of gas imaging techniques in leak quantification. In these cases, the covariance error term is responsible for between a quarter to a third of the projection uncertainties depending on the observed downwind distance from the leak source. Furthermore, we found that increasing the acquisition time by a few seconds (∼5 s) can cause substantial (>50%) decreases in the projection uncertainties leading to much more robust estimates for the leak rate. The employment of long acquisition times and imaging far away from leak sources may prove difficult to achieve in real life releases. In particular, variations in wind direction that are more likely to occur over longer periods can impede the emission estimation process by moving the plume away from the view of the camera. Meanwhile, at farther distances downwind of leak sources the gas concentrations within the plume are likely to drop below the detection limit of gas imaging cameras which can lead to underestimation of the leak rates. Consequently, for practical applications, we suggest the use of multiple control volumes at varying distances coupled with the longest acquisition times as allowed by the environmental conditions.

Altogether, the remote sensing approach based on the use of gas imaging technology is a promising technique that has the capacity for accurate leak quantification. This approach allows for non-intrusive leak quantification without the need for additional equipment for wind measurements. To the best of our knowledge, previous studies on leak quantification via gas imaging have only used a single control volume close to the source, whereas our findings suggest that estimates can be improved by employing multiple control volumes [21,22]. With the development of new hyperspectral cameras recording images at high spatial and temporal resolutions and more efficient velocimetry algorithms, it is expected that the accuracy and speed in leak quantification can further improve as long as projection uncertainties are kept in check with our suggested guidelines.

## Figures and Tables

**Table 1:** Summary of parameters used in LES.

Name	Value
Computational domain size ($x_{max},y_{max},z_{max})$	60, 60, 20 (m)
Computational grid size (Δx,Δy,Δz)	0.469, 0.469, 0.188 (m)
Height of source ($z_{p}$)	2.25 (m)
Sampling frequency ($f_{s}$)	1 (Hz)
Sampling duration ($T_{s}$)	900 (s)
Downwind distance of intersects ($x_{m}$)	4.7, 7.0, 8.9, 17.9, 26.8 (m)
Normalized downwind distance of intersects ($x_{m}/z_{p}$)	2.1, 3.1, 4.0, 8.0, 11.9 (-)

## Data Availability

The Large Eddy Simulation data presented in this study are available on request from the corresponding author.
